# Activation of the NLRP3 inflammasome by human adenovirus type 7 L4 100-kilodalton protein

**DOI:** 10.3389/fimmu.2024.1294898

**Published:** 2024-04-17

**Authors:** Yali Duan, Yun Zhu, Linlin Zhang, Wei Wang, Meng Zhang, Jiao Tian, Qi Li, Junhong Ai, Ran Wang, Zhengde Xie

**Affiliations:** ^1^ Beijing Key Laboratory of Pediatric Respiratory Infectious Diseases, Key Laboratory of Major Diseases in Children, Ministry of Education, National Clinical Research Center for Respiratory Diseases, Laboratory of Infection and Virology, Beijing Pediatric Research Institute, Beijing Children’s Hospital, Capital Medical University, National Center for Children's Health, Beijing, China; ^2^ Research Unit of Critical Infection in Children, 2019RU016, Chinese Academy of Medical Sciences, Beijing, China; ^3^ Department of Infectious Diseases, Beijing Children’s Hospital, Capital Medical University, National Center for Children’s Health, Beijing, China; ^4^ Department of Pediatrics, Beijing Jingmei Group General Hospital, Beijing, China; ^5^ Department of Pediatric Rehabilitation, Beijing Boai Hospital, School of Rehabilitation Medicine, Capital Medical University, China Rehabilitation Research Center, Beijing, China

**Keywords:** human adenovirus type 7, NLRP3, inflammasome, NACHT, LRR

## Abstract

Human adenovirus type 7 (HAdV-7) is a significant viral pathogen that causes respiratory infections in children. Currently, there are no specific antiviral drugs or vaccines for children targeting HAdV-7, and the mechanisms of its pathogenesis remain unclear. The NLRP3 inflammasome-driven inflammatory cascade plays a crucial role in the host’s antiviral immunity. Our previous study demonstrated that HAdV-7 infection activates the NLRP3 inflammasome. Building upon this finding, our current study has identified the L4 100 kDa protein encoded by HAdV-7 as the primary viral component responsible for NLRP3 inflammasome activation. By utilizing techniques such as co-immunoprecipitation, we have confirmed that the 100 kDa protein interacts with the NLRP3 protein and facilitates the assembly of the NLRP3 inflammasome by binding specifically to the NACHT and LRR domains of NLRP3. These insights offer a deeper understanding of HAdV-7 pathogenesis and contribute to the development of novel antiviral therapies.

## 1 Introduction

Human adenovirus (HAdV) is a double-stranded DNA virus without an envelope, classified within the family *Adenoviridae*, the genus *Mastadenovirus*. The genome of HAdV is about 36 kb in size and consists of five early transcription units (E1A, E1B, E2, E3, E4), four intermediate transcription units (IX, IVa2, L4 intermediate, and late E2) during the initiation of viral DNA replication, and one late transcription unit. Upon processing, it produces five late mRNA families (L1–L5); it also encodes over 40 structural or non-structural proteins.

Based on immunological, biological and biochemical characteristics, HAdV is divided into seven groups (A–G) and 113 serotypes/genotypes have been identified (http://www.advhwg.gmu.edu/). Each type of HAdV has its own tissue tropism and can lead to different diseases (1). Within the HAdV-B group, HAdV-3, -7, -14, and -55 are common pathogens that cause respiratory diseases. HAdV-7 has a longer detoxification time compared with other types (2), resulting in more severe clinical symptoms, higher mortality rate (3–6), and more challenging treatment, which may also lead to long-term pulmonary sequelae (7). In China, especially in Northern China, HAdV-7 is one of the most dominant types responsible for respiratory infections in children. From 2007 to 2013, In children, HAdV-7 infections represented between 28.6% and 49% of acute respiratory diseases attributed to HAdV (4, 8, 9). In 2009, an outbreak of infant pneumonia caused by HAdV-7 occurred in Shaanxi Province, resulting in the death of one infant (10). In the spring and summer of 2019, a significant outbreak of respiratory infection in children caused by HAdV, primarily HAdV-7, took place in southern China, with 51.4% of cases categorized as severe (11). Nonetheless, there is currently no specific antiviral therapy and prevention for HAdV-7 infection and the underlying pathogenic mechanisms are not fully understood.

The innate immune system acts as the primary defense mechanism against viral infections. Pattern-recognition receptors (PRRs) are pivotal in identifying viruses and triggering the activation of the innate immune response (12). One significant component of this defense mechanism is the inflammasome, a protein complex formed within host cells in response to various stimuli, such as pathogens or injury. The inflammasome consists of recognition molecules, adaptor molecules, and effector molecules. The molecules involved in recognition are NOD-like receptors (NLRs), and the adaptor molecules predominantly consist of apoptosis-associated speck-like protein containing a CARD (ASC). The effector molecules, responsible for executing the response, belong to the caspase family and include caspase-1 or caspase-4/5 in humans (13).

The NLRP3 protein comprises three essential domains: the PYD domain, which facilitates interaction with downstream signaling proteins; the NACHT domain, common to all NLRs family members and crucial for inflammasome assembly; and the LRR domain, responsible for ligand sensing and autoregulation. Upon receiving stimulating signals (such as ATP), the NACHT domain interacts and self-oligomerizes, forming complexes that trigger the self-activation of caspase-1. Consequently, pro-IL-1β precursor is cleaved, and IL-1β is released into the extracellular medium, leading to cell pyroptosis (12). Activation of the inflammasome leads to the secretion of inflammatory factors and cellular demise, which collectively combat viral infections. In the context of HAdV-7 infection, our previous research has shown that it can activate the NLRP3 inflammasome (14). However, the exact underlying mechanism remains incompletely understood.

Studies have investigated the inflammasome activation caused by HAdV infection, with a particular focus on HAdV-5 in the HAdV-C group. Researchers have found that when HAdV-5 infects human macrophages, it leads to the release of double-stranded DNA. This DNA release triggers Toll-like receptor 9 (TLR9) activation in the endosomal membrane, leading to an increase in the expression of NLRP3, pro-caspase-1, and pro-IL-1β mRNA. Additionally, lysosomal rupture induced by HAdV-5 infection causes the release of cathepsin B and reactive oxygen species (ROS), which subsequently enhance the formation and activation of the NLRP3 inflammasome, culminating in the secretion of IL-1β (15). Furthermore, HAdV-5 has been found to activate the AIM2 inflammasome through a cell-dependent process involving protein VI-mediated endosomal escape. This activation triggers IL-1β secretion and leads to Gasdermin D cleavage, ultimately resulting in pyroptosis of monocyte-derived dendritic cells (16).

The activation of the NLRP3 inflammasome by HAdV-5 may be influenced by various factors such as its colocalization with lysosomes and difference in viral membrane penetration mechanism (17). It’s important to note that different types of HAdV exhibit distinct immunophenotypes and biological properties, which can impact their endosome transport processes and, consequently, the mechanisms of inflammasome activation. For example, upon entering the cell, HAdV-5 rapidly transports to the nucleus through early endosomes, whereas HAdV-35 and HAdV-26 tend to accumulate in late endosomes for a longer duration after infection, resulting in elevated secretion of proinflammatory cytokines and chemokines (18).

HAdV-5 and HAdV-7, belonging to different groups, exhibit significant differences in biological characteristics, including receptor utilization and endosomal transport processes (19–21). As a result, their mechanisms of NLRP3 inflammasome activation are not entirely consistent. Previous findings indicate that HAdV-7 infection induces an up-regulation of NLRP3, caspase-1, pro-IL-1β mRNA expression by activating TLR4, and subsequently triggers NLRP3 inflammasome activation through K^+^ efflux and lysosomal disruption (22). However, further studies on this matter remain scarce. To address this knowledge gap, our study aims to delve deeper into the molecular mechanisms by which HAdV-7-encoded viral proteins activate the NLRP3 inflammasome. By doing so, we aim to enhance our comprehension of the pathogenesis of HAdV-7 infection, uncover novel therapeutic approaches, and identify potential molecular targets for the development of antiviral drugs or vaccines.

## 2 Materials and methods

### 2.1 Cell lines and cultures

The HEK293T cell line, derived from human embryonic kidney cells, was acquired from the American Type Culture Collection (ATCC) located in Manassas, VA, USA. The A549 human non-small-cell lung cancer cell line and HeLa human epithelial carcinoma cell line were procured from the Chinese Academy of Medical Sciences. Human monocytic cells (THP-1) were kindly provided by Dr. Xia Xiao from the Institute of Pathogen Biology, Chinese Academy of Medical Sciences and Peking Union Medical College, Beijing, China. A549, HEK293T and HeLa cell lines were cultured in Dulbecco’s modified Eagle medium (DMEM) purchased from Gibco in Grand Island, NY, USA. The medium was enriched with 10% fetal bovine serum (FBS), 100 U/mL penicillin, and 100 μg/mL streptomycin sulfate. THP-1 cells were cultivated in RPMI 1640 medium, additionally supplemented with 10% heat-inactivated FBS, 100 U/mL penicillin, and 100 μg/mL streptomycin sulfate. All cells were sustained in a humidified incubator at 37°C under a 5% CO_2_ atmosphere.

### 2.2 Plasmid

The 47 genes of HAdV-7 (GenBank accession number MG923582) were synthesized by SinoGenoMax Co., Ltd, Beijing, China, and cloned into the pcDNA3.0 (+)-eGFP expression vector with a Flag-tag. Moreover, pCMV6-NLRP3, pCA7-ASC, pCA7-pro-Casp-1, and pCA7-pro-IL-1β plasmids were graciously supplied by Prof. Xiaobo Lei from the Institute of Pathogen Biology, Chinese Academy of Medical Sciences and Peking Union Medical College, Beijing, China. Furthermore, GV712-NLRP3, GV712-NLRP3 (PYD), GV712-NLRP3 (NACHT), GV712-NLRP3 (LRR), GV366-100 kDa-HA, and pGEX-100 kDa were constructed by Shanghai Genechem Co.,Ltd.

### 2.3 Lentivirus

The 100 kDa-lentivirus and control CT-lentivirus were prepared by Shanghai Genechem Co.,Ltd. The titers of the 100 kDa-lentivirus and control CT-lentivirus were determined to be 3 × 10^8^/mL and 2 × 10^8^/mL, respectively, using the qPCR method.

### 2.4 Reagents

Lipopolysaccharide (LPS), adenosine triphosphate (ATP), Nigericin, and ProLong™ Diamond Artifade Mountant with Dapi were purchased from InvivoGene Biotech Co., Ltd. (San Diego, CA, USA). PMA was sourced from Sigma-Aldrich (St. Louis, MO, USA). RPMI-1640, DMEM, and FBS were procured from Gibco (Grand Island, NY, USA).

The rabbit anti-GFP-Tag antibody (SAB4301138) and mouse anti-HA-Tag antibody (H9658), along with EZview™ Red anti-HA beads (E6779) and anti-FLAG^®^ M2 beads (A2220), were obtained from Sigma (St Louis, MO, USA). Monoclonal rabbit anti-NLRP3 (D2P5E), rabbit anti-DYKDDDDK/FLAG Tag antibody (14793), monoclonal rabbit anti-IL-1β (D3U3E), and monoclonal rabbit anti-caspase-1 (2225) were obtained from Cell Signaling Technology (Beverly, MA, USA). Monoclonal mouse anti-ASC (sc-271054) (1:500) and polyclonal rabbit anti-IL-1β (sc-7884) (1:500) were purchased from Santa Cruz Biotechnology (Santa Cruz, CA, USA).

### 2.5 Enzyme-linked immunosorbent assay (ELISA)

IL-1β concentrations in culture supernatants were measured using the ELISA Kit (Excell Bio, Shanghai, China) according to the manufacturer’s protocol.

### 2.6 THP-1 macrophage stimulation

THP-1 cells were induced to differentiate into macrophages through treatment with 100 ng/mL PMA for 16–18 h, followed by culturing them for an additional 24 h without PMA. Subsequently, the differentiated cells were stimulated with LPS, Nigericin, or ATP. Culture supernatants were collected to measure IL-1β levels using ELISA, while the cells were harvested for immunoblot analysis.

### 2.7 Western blot analysis

Whole-cell lysates from HEK293T and PMA-differentiated THP-1 cells were prepared using NP-40 lysis buffer (Beyotime, P0013F). The protein concentration was determined using the Bradford assay (Bio-Rad, Hercules, CA, USA). Cell lysates (30 μg) from cultured cells were subjected to electrophoresis on a 10% or 12% SDS-polyacrylamide gel electrophoresis (PAGE) gel and then transferred onto a polyvinylidene difluoride (PVDF) membrane (Millipore, MA, USA). Following this, the PVDF membranes were blocked with 5% skim milk in PBS with 0.1% Tween 20 (PBST) prior to antibody incubation. Protein bands were virsulized using an enhanced chemiluminescence (ECL) Kit (Beyotime, P0018).

### 2.8 Co-immunoprecipitation assays (Co-IP)

HEK293T cells reaching 70–80% confluency in 6 cm dishes were co-transfected with the specified plasmids and incubated for 24–48 h. Similarly, THP-1-100 kDa stable cells were also transfected as described. The transfected HEK293T cells or THP-1-100 kDa stable cells were then lysed in NP-40 lysis buffer (Beyotime, P0013F). The lysates were immunoprecipitated using EZview™ Red anti-HA beads (Sigma, E6779) or anti-FLAG^®^ M2 beads (Sigma, A2220). The immunoprecipitates were washed extensively with the appropriate lysis buffer, boiled in protein loading buffer, and subjected to analysis via western blotting.

### 2.9 Confocal microscopy

HEK293T, HeLa, and A549 cells cultured on sterile coverslips were transfected with the specified plasmids when reaching 30–40% confluence and incubated for 24 h. PMA-differentiated THP-1-CT or THP-1-100 kDa stable cells were also cultured on sterile coverslips for 24 h. These cells were then fixed with 4% paraformaldehyde for 30 min, followed by three washes with ice-cold PBS containing 0.1% bovine serum albumin (BSA). Subsequently, the cells were permeabilized and blocked with solution (Beyotime, P0260) at room temperature for 20 min. Subsequently, the cells were incubated overnight at 4 °C with the appropriate primary antibody, followed by staining with fluorochrome-conjugated secondary antibodies (Alexa Fluor 488, 555, and 647; 1:500 dilution; Cell Signal Technology, 4408S, 4413S, and 4410S). The nuclei were counterstained with antifade Mounting Medium containing DAPI (4’,6-diamidino-2-phenylindole), and the cells were washed three times. Subsequently, the cells were examined under a confocal fluorescence microscope (Leica, SP8).

### 2.10 Statistical analyses

All experiments were reproducible and repeated at least three times, yielding similar results. Statistical analysis was conducted using GraphPad Prism 7, employing the *t*-test for two groups and one-way analysis of variance for multiple groups. Statistical significance was considered when ^*^ *P* ≤ 0.05, ^**^ *P* ≤ 0.01, and ^***^ *P* ≤ 0.001.

## 3 Results

### 3.1 The 100 kDa protein, encoded by the HAdV-7 L4 gene, plays a pivotal role in activating the NLRP3 inflammasome, thereby promoting the production of IL-1β

**Figure 1:**
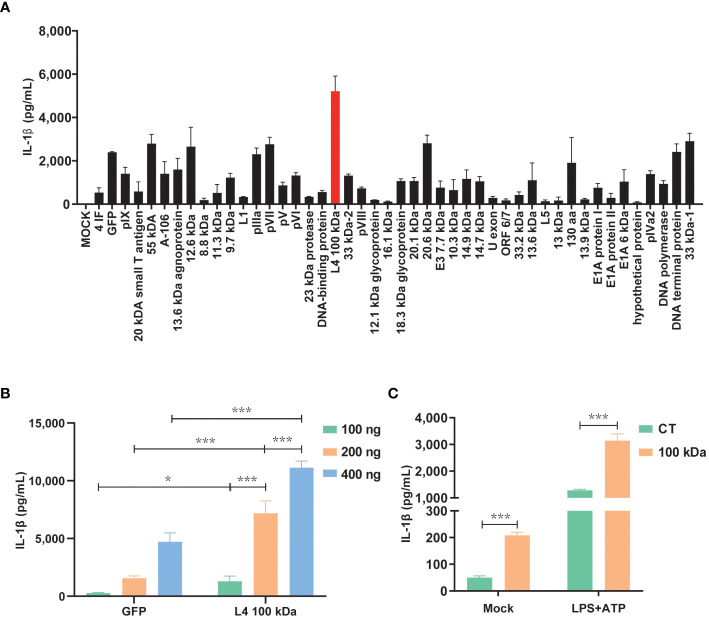
Activation of the NLRP3 inflammasome by HAdV-7 100 kDa protein. **(A, B)** HEK293T cells were co-transfected with plasmids encoding NLRP3, ASC, pro-caspase-1, and pro-IL-1β. Subsequently, the cells were transfected with plasmids encoding 47 different HAdV-7 proteins in **(A)** or with varying concentrations of pcDNA3.0-100 kDa in **(B)**. IL-1β levels in the supernatants were quantified using ELISA. **(C)** THP-1 cells were infected with CT-lentivirus or 100 kDa-lentivirus (encoding the 100 kDa protein), differentiated into macrophages through PMA stimulation, and then treated with LPS (1 μg/ml) for 1 h plus ATP (5 mM) for 2 h. IL-1β levels in the supernatants were assessed by ELISA. Data is representative of three independent experiments, with one representative experiment presented. Error bars indicate the standard deviation (SD) of technical triplicates. Values are expressed as mean± standard error of the mean (SEM). ^*^, *P* < 0.05; ^***^, *P* < 0.001.

Previous studies have demonstrated that swift activation of the NLRP3 inflammasome may not rely on prior inflammasome preactivation (23). Furthermore, inhibiting viral protein synthesis has been shown to impede the activation of NLRP3 inflammasome by viruses (24). To investigate this further, we constructed eukaryotic expression plasmids encoding 47 HAdV-7 viral proteins and assessed their expression efficiency in cells. Subsequently, the HEK293T cells were co-transfected with plasmids encoding three constituents (NLRP3, ASC, and pro-Caspase-1) of the NLRP3 inflammasome and pro-IL-1β, along with plasmids encoding the 47 viral proteins, respectively. We then measured the IL-1β expression level in the cell supernatant using ELISA. Remarkably, the results revealed that the group transfected with the 100 kDa protein exhibited the highest IL-1β expression level compared to the control group (consisting of empty vector plus NLRP3 inflammasome component co-transfection group, **Figure 1A**). Additionally, the effect was concentration-dependent (**Figure 1B**), and the observed disparity was statistically significant (*P*<0.05).

To further corroborate the role of the 100 kDa protein in NLRP 3 inflammasome activation, we performed lentivirus transfection of THP-1 cells to establish cell lines stably expressing the 100 kDa protein. Subsequent experiments confirmed that under LPS+ATP stimulation, IL-1β secretion was significantly enhanced by the presence of the 100 kDa protein (**Figure 1C**). These findings provide evidence supporting the involvement of the 100 kDa protein in NLRP3 inflammasome activation and the subsequent promotion of IL-1β production.

### 3.2 The 100 kDa protein effectively activates the NLRP3 inflammasome through its interaction with NLRP3 inflammasome components

**Figure 2:**
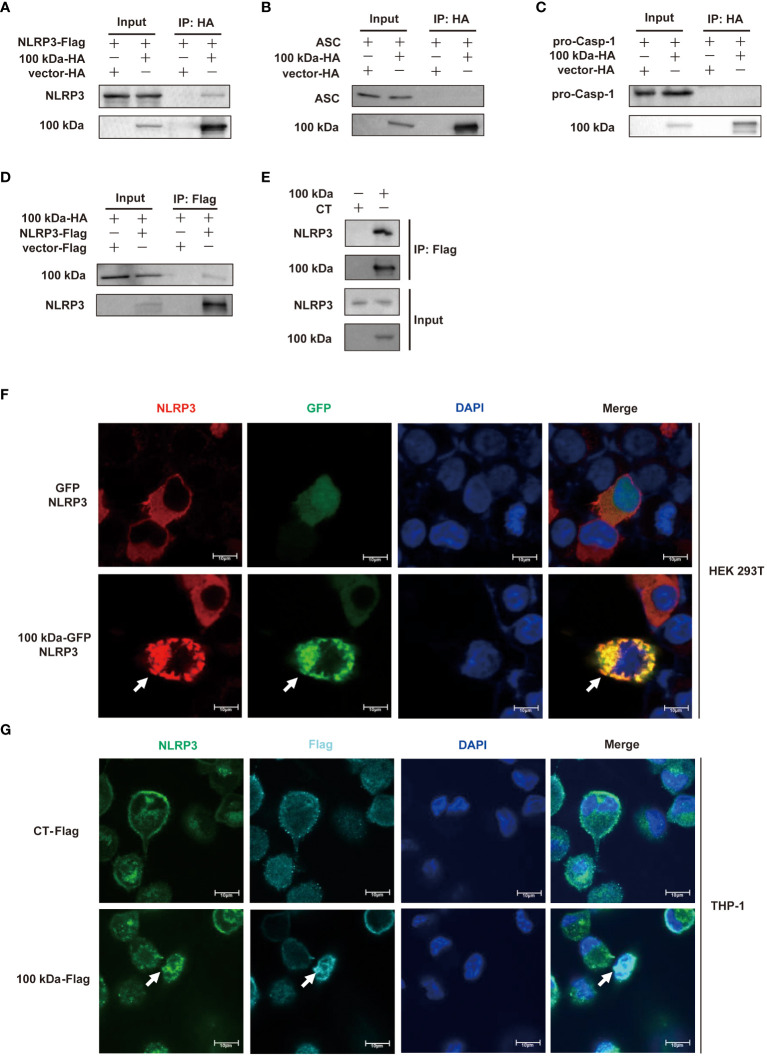
Interaction of HAdV-7 100 kDa protein with NLRP3. **(A–D)** HEK293T cells were co-transfected with 100 kDa-HA and NLRP3-Flag in **(A)**, 100 kDa-HA and ASC in **(B)**, and 100 kDa-HA and pro-caspase-1 in **(C)** and 100 kDa-HA and NLRP3-Flag in **(D)**. Cell lysates were prepared and subjected to immunoprecipitation (IP) using EZview™ Red anti-HA beads **(A–C)** or anti-FLAG^®^ M2 beads **(D)**, followed by immunoblotting with anti-HA or anti-NLRP3, anti-ASC, and anti-caspase-1 antibody. **(E)** THP-1 macrophages were stably infected with CT-Lentivirus or 100 kDa-Lentivirus, differentiated into macrophages by PMA stimulation with for 48 (h) Cell lysates were immunoprecipitated using anti-FLAG^®^ M2 beads and analyzed with anti-NLRP3 and anti-Flag antibody. **(F)** HEK293T cells were transfected with pCA7-NLRP3, GFP-100 kDa/GFP for 24 h. Subcellular localizations of NLRP3 (red), nucleus marker DAPI (blue), and GFP-100 kDa/GFP (green) was visualized using confocal microscopy. **(G)** PMA-differentiated THP-1 macrophages were stably infected with CT-lentivirus or 100 kDa-lentivirus. Subcellular localizations of NLRP3 (green), nucleus marker DAPI (blue), and 100 kDa-Flag/Flag (light blue) were visualized using confocal microscopy. Scale bar represents 10 μm.

To elucidate the underlying mechanism of NLRP3 inflammasome activation by the 100 kDa protein, we conducted thorough investigations. It has been previously observed that viral proteins have the capacity to trigger the activation of the NLRP3 inflammasome by interacting with its components. In our study using HEK293T cells, we found that the 100 kDa protein specifically bound to NLRP3 but did not interact with ASC or pro-caspase-1 (**Figures 2A–D**). This finding suggested that the 100 kDa protein was associated with NLRP3, but not with ASC or pro-caspase-1.

Moreover, our observations in THP-1 cells expressing the 100 kDa protein further supported this interaction as we found that the 100 kDa protein also bound to NLRP3 in these cells (**Figure 2E**).

To gain further insight into the subcellular localization of the 100 kDa-NLRP3 interaction, we co-transfected pCMV6-NLRP3 plasmid with the 100 kDa-GFP plasmid into HEK293T cells and visualized the localization of these proteins using confocal microscopy. Our results clearly showed that in the absence of 100 kDa, NLRP3 was broadly distributed throughout the cytoplasm (**Figure 2F**, first row). However, in the presence of 100 kDa, NLRP3 and 100 kDa exhibited co-localization, forming distinct spots in the cytoplasm (**Figure 2F**, second row). Similar observations were made in THP-1 cells overexpressing the 100 kDa protein, further confirming this interaction (**Figure 2G**).

Based on these results, we conclude that the 100 kDa protein activates the NLRP3 inflammasome by specifically interacting with the NLRP3 protein. This interaction is likely a crucial step in initiating the NLRP3 inflammasome signaling cascade, leading to the production of IL-1β.

### 3.3 The crucial binding of the 100 kDa to the NLRP3 protein involves its interaction with the NACHT and LRR domains

The NLRP3 protein is comprised of three key domains: PYD, NACHT, and LRR domains (12). The PYD domain of NLRP3 protein is known to interact with ASC protein. To identify the specific domain of NLRP3 protein responsible for its interaction with the 100 kDa protein, we constructed plasmids encoding the individual PYD, NACHT, and LRP domains.

**Figure 3:**
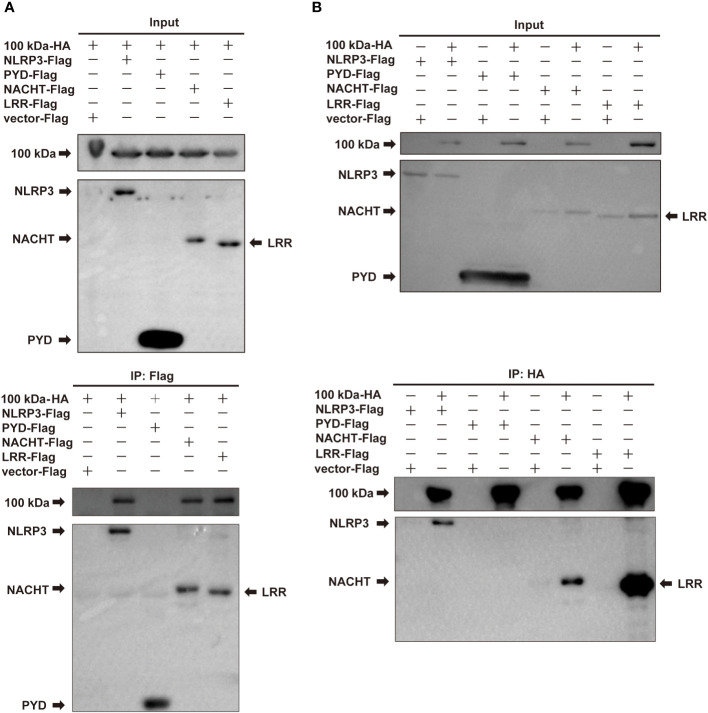
Interaction of HAdV-7 100 kDa with NLRP3 domains. **(A, B)** HEK293T cells were co-transfected with 100 kDa-HA and NLRP3-Flag, PYD-Flag, NACHT-Flag, or LRR-Flag. Cell lysates were prepared and subjected to immunoprecipitation (IP) using EZview™ Red anti-HA beads or anti-FLAG^®^ M2 beads, followed by immunoblotting with anti-HA or anti-Flag antibodies.

Subsequently, we constructed four plasmids expressing NLRP3, PYD, NACHT, and LRR domains, which were then co-transfected with the plasmid encoding the 100 kDa protein into HEK293T cells for co-IP experiments. The results of these experiments revealed that the 100 kDa protein precipitated with NLRP3, NACHT and LRR domains, but not with PYD domain (**Figures 3A, B**), indicating that the interaction between the 100 kDa protein and NLRP3 occurs specifically through the NACHT and LRR domains. Our findings point towards the significance of the NACHT and LRR domains in facilitating the interaction between the 100 kDa protein and the NLRP3 protein.

### 3.4 The 100 kDa exhibits direct binding to the NLRP3 protein and participates in the assembly of the NLRP3 inflammasome

**Figure 4:**
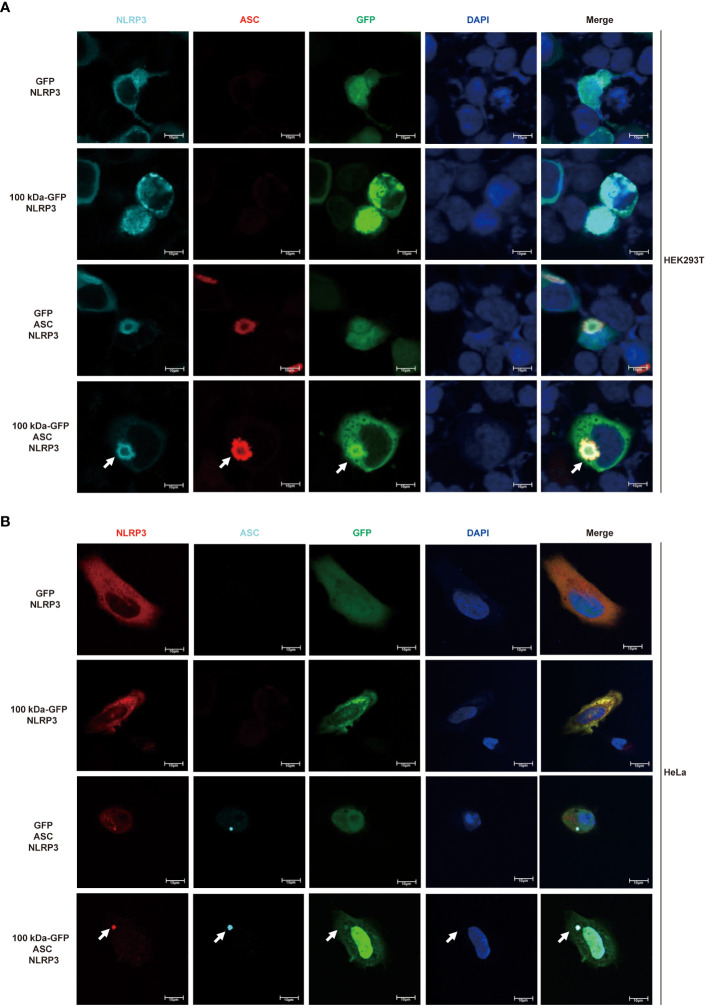
Participation of HAdV-7 100 kDa in NLRP3 inflammasome assembly. **(A)** HEK293T cells and **(B)** HeLa cells were co-transfected with different combinations of constructs, including GFP/NLRP3, 100 kDa-GFP/NLRP3, GFP/NLRP3/ASC, and 100 kDa-GFP/NLRP3/ASC for 24 (h) Subcellular localization of NLRP3, ASC, 100 kDa-GFP or GFP, and nucleus marker DAPI were visualized using confocal microscopy.

Based on the experimental findings mentioned earlier, we speculated that the 100 kDa protein plays a crucial role in the assembly of the inflammasome after HAdV-7 infection by directly binding to the NLRP3 protein. To further explore the impact of the 100 kDa protein on the NLRP3 inflammasome, we utilized confocal microscopy to observe the localization of these proteins in HEK293T cells after co-transfection of plasmids encoding GFP, 100 kDa-GFP, NLRP3 and/or ASC. Previous studies have indicated that the formation of spots of NLRP3 protein in the cytoplasm serves as a marker for the formation of the inflammasome complex (25). When both 100 kDa and ASC were absent, NLRP3 was widely distributed throughout the cytoplasm (**Figure 4A**, first line). In the presence of 100 kDa but absence of ASC, NLRP3 co-localized with 100 kDa (**Figure 4A**, second line) and exhibited a spotted or annular pattern in the cytoplasm. Conversely, in the absence of 100 kDa but presence of ASC, NLRP3 co-localized with ASC and showed a spotted or circular aggregation in the cytoplasm (**Figure 4A**, third line). Importantly, when 100 kDa and ASC were both present, NLRP3, 100 kDa, and ASC co-localized (**Figure 4**, fourth line) and formed a ring-like structure in the cytoplasm of transfected cells. Our findings suggest that the 100 kDa protein plays an important role in assembling the NLRP3 inflammasome by binding to NLRP3 to form the “100 kDa-NLRP3-ASC” complex.

**Figure 5:**
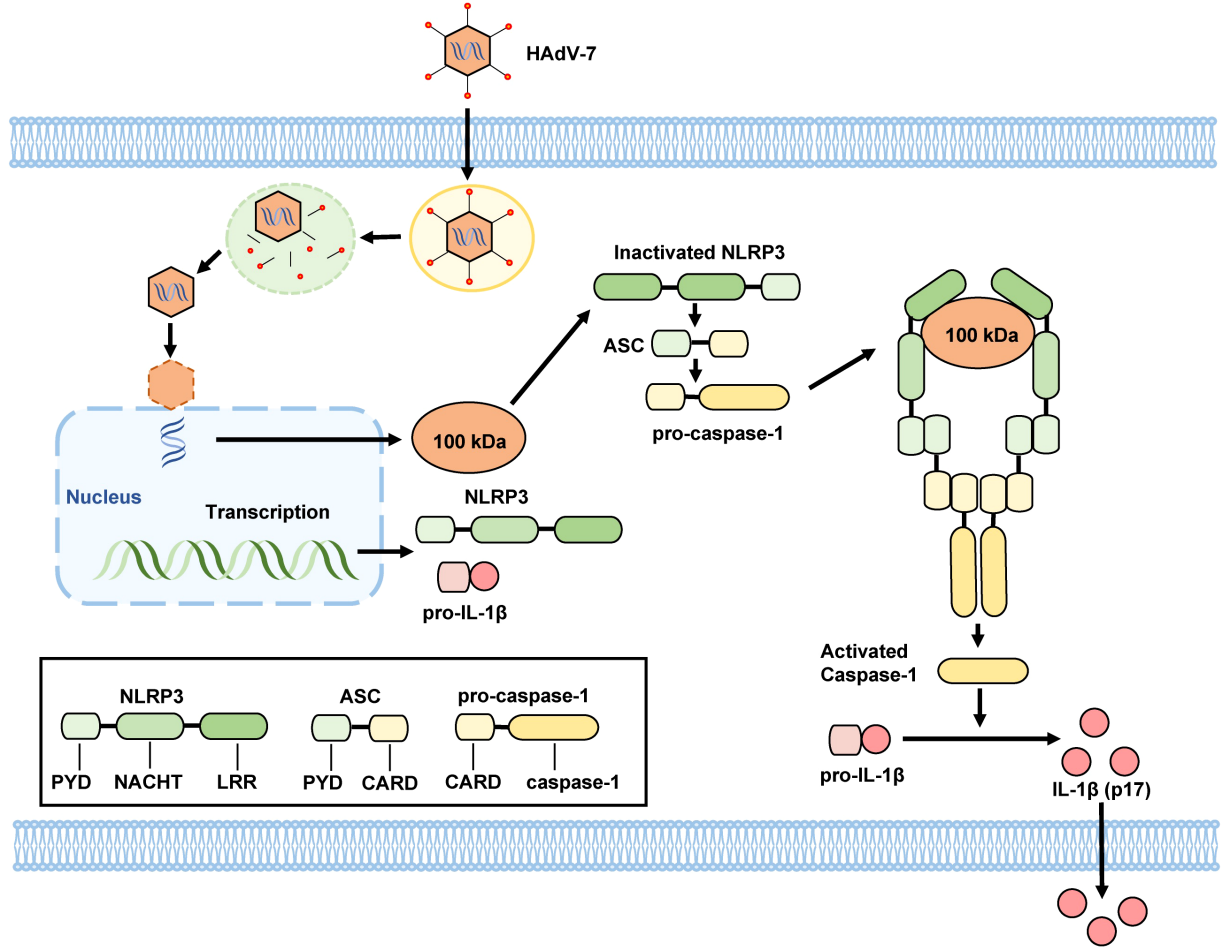
Proposed mechanism by HAdV-7 infection-induces inflammatory responses. The 100 kDa protein encoded by HAdV-7 binds to NACHT and LRR domains of the NLRP3 protein, facilitating the interaction of the PYD domain of NLRP3 with the ASC. Subsequently, ASC binds to pro-caspase-1 through its CARD domain, leading to the self-cleavage of pro-caspase-1 and the formation of caspase-1 (p20/p10). Caspase-1 (p20/p10) then cleaves pro-IL-1β, resulting in the formation of IL-1β, which is subsequently secreted from the cell.

Drawing on existing literature and our results, we propose a mechanism for the activation of the NLRP3 inflammasome in HAdV-7-infected THP-1 cells: the 100 kDa protein encoded by HAdV-7 attaches to the NACHT and LRR domains of NLRP3 protein, facilitating the interaction of the PYD domain of NLRP3 with ASC. Consequently, ASC binds to pro-caspase-1 through its CARD domain, leading to the self-cleavage of pro-caspase-1 and the formation of caspase-1 (p20/p10). Then, caspase-1 (p20/p10) cleaves pro-IL-1β, resulting in the formation of IL-1β, which is then secreted from the cell (**Figure 5**). This proposed mechanism illuminates the process of NLRP3 inflammasome activation triggered by HAdV-7 infection in THP-1 cells.

## 4 Discussion

NLRP3 inflammasome plays a crucial role in host resistances to DNA virus infections. Previous studies have demonstrated that Herpes simplex virus type 1 (HSV-1) induces the activation of the NLRP3 inflammasome in mouse glial cells, resulting in the production of mature IL-1β and release of caspase-1 (p10), thus providing protection against HSV-1 infection (26). In our previous study, we observed that HAdV-7 infection of THP-1 cells could also activate the NLRP3 inflammasome and induce an inflammatory response (14). Building upon these findings, the current study further reveals that the 100 kDa protein, encoded by the L4 gene of HAdV-7, plays a significant role in NLRP3 inflammasome activation. Specifically, it contributes to inflammasome assembly through interaction with the NACHT and LRR domains of the NLRP3 protein. This discovery represents a novel advancement in our understanding of HAdV-induced inflammasome activation and offers valuable insights for the development of antiviral drugs.

The 100 kDa protein plays a vital role in HAdV replication by modulating cellular mechanisms. It facilitates the translation and nuclear transport of numerous viral protein products, leading to their accumulation and assembly into capsid (27–33). During the late stages of HAdV replication, the 100 kDa protein interacts with hexon monomers of major capsid proteins, forming a trimer structure (34–36). Moreover, the 100 kDa protein acts as a molecular chaperone for Hexon trimers and is involved in nuclear transport and capsid assembly (37). Additionally, this study has revealed a new function of the 100 kDa protein as an inhibitor of Granzyme B (GrB), which binds to GrB and effectively inhibits its activity, thus preventing cytotoxic lymphocyte-induced apoptosis of target cells (38). However, the role of the 100 kDa protein in the modulation of the NLRP3 inflammasome has not been reported before.

In the absence of any stimulus signal, NLRP3 protein was observed to be homooligomerized. However, upon sensing an activation signal, NLRP3 protein interacted with ASC, leading to the recruitment of pro-casepase-1 through the CARD domain of ASC protein, thereby generating active caspase-1 (p20/p20) via self-cleavage (39, 40). While direct interactions between 100 kDa and ASC or pro-casepase-1 were not observed, it was found that 100 kDa interacted with NLRP3 and associated with ASC and pro-casepase-1. Confocal microscopy analysis demonstrated that in the absence of 100 kDa protein, NLRP3 and ASC were dispersed in the cytoplasm. When only 100 kDa and NLRP3 were present, they aggregated to form spots. Interestingly, when all three proteins, 100 kDa, NLRP3, and ASC, were present simultaneously, they formed spots or ring-like structures and co-localized, characteristics of an inflammasome (25). Similar structures, termed “viral protein-NLRP3-ASC”, have been observed in inflammasome activated by viral proteins encoded by Enterovirus 71 (41) and Zika virus (42). Hence, these results suggested that the 100 kDa protein is involved in assembling the NLRP3 inflammasome by binding to NLRP3, forming the “100 kDa-NLRP3-ASC” structure.

However, it is essential to acknowledge the limitations of this study. Firstly, the mechanism of 100 kDa protein activating the NLRP3 inflammasome was primarily explained at the cellular level. Further verification using appropriate animal models is necessary to establish its effectiveness *in vivo*. Secondly, while this study elucidated the protein-protein interactions during NLRP3 inflammasome activation by HAdV-7 infection, the impact on virus replication in HAdV-7-infected cells was not explored in depth.

In conclusion, considering the economic burden and health threats posed by adenovirus pneumonia, delving into its mechanisms holds profound significance (43). This study reveals a novel regulatory mechanism involving the 100 kDa protein encoded by HAdV-7 in the activation of the NLRP3 inflammasome. By binding to the NACHT and LRR domains of NLRP3, the 100 kDa protein facilitates the formation of a “100 kDa-NLRP3-ASC” ring structure, thus promoting the assembly of the NLRP3 inflammasome. This mechanism highlights the potential significance of 100 kDa in modulating inflammatory responses during HAdV-7 infection, paving the way for potential treatment targeting HAdV-7 infection. However, further research is required to fully understand the therapeutic implications of these findings.

## Data availability statement

The raw data supporting the conclusions of this article will be made available by the authors, without undue reservation.

## Author contributions

YD: Conceptualization, Investigation, Writing – original draft, Writing – review & editing. YZ: Methodology, Writing – review & editing. LZ: Investigation, Methodology, Writing – review & editing. WW: Writing – review & editing. MZ: Investigation, Methodology, Writing – review & editing. JT: Investigation, Methodology, Writing – review & editing. QL: Investigation, Software, Writing – review & editing. JA: Investigation, Writing – review & editing. RW: Writing – original draft, Writing – review & editing. ZX: Conceptualization, Funding acquisition, Resources, Writing – original draft, Writing – review & editing.
